# Effect of temperature and humidity on dynamics and transmission of *Pseudomonas amygdali* pv. *lachrymans* aerosols

**DOI:** 10.3389/fpls.2023.1087496

**Published:** 2023-02-03

**Authors:** Ali Chai, Lifang Yuan, Xin Li, Lei Li, Yanxia Shi, Xuewen Xie, Baoju Li

**Affiliations:** ^1^ Institute of Vegetables and Flowers, Chinese Academy of Agricultural Sciences, Beijing, China; ^2^ Shandong Academy of Agricultural Sciences, Shandong Academy of Grape, Jinan, Shandong, China

**Keywords:** temperature, relative humidity, bioaerosol, *Pseudomonas amygdali* pv. *lachrymans*, cucumber angular leaf spot disease

## Abstract

Cucumber angular leaf spot (ALS) disease, caused by *Pseudomonas amygdali pv. lachrymans (Pal)*, is an emerging disease with a high incidence that causes severe damage to cucumber worldwide. Bacterial aerosols play a crucial role in the epidemiology of greenhouse ALS disease. However, little is known about the influence of temperature and relative humidity (RH) on the dynamics of *Pal* in aerosols. A study was conducted to investigate the relationships between the concentration of *Pal* aerosols and their dependence on temperature and RH in aerosol chambers and greenhouses. The results demonstrated that temperature and RH are both significant factors influencing the release amount, survival time and infectivity of *Pal* in aerosols, while RH has a greater influence on particle size than temperature across the range of conditions tested. The release amount and survival time of *Pal* in aerosols under high RH (95%) and low temperature (≤ 25°C) conditions were significantly higher than those under low RH (35%) and high temperature (35°C) conditions. The highest release amount of *Pal* aerosol (96 CFU/m^3^) and highest survival rate (98.41%) were found at 18°C and 95% RH, while the highest disease index (DI = 60.9) caused by *Pal* aerosol was found at 25°C and 95% RH. In addition, *Pal* aerosols presented a larger diameter (4.7->7.0 μm) under high RH (95% RH) than under dry conditions (≤ 65% RH). These findings will play a crucial role in elucidating the influence of environmental parameters on the dynamics and transmission of *Pal* in aerosols. Based on our findings, preliminary recommendations for controlling airborne *Pal* spread involve controlling air temperature and RH, which will contribute to the effective alleviation and control of cucumber ALS disease.

## 1 Introduction

Cucumber is one of the most important vegetable crops in many countries worldwide. Statistically, China is the largest cucumber producer in the world, with 1.27 Mha of cultivation area and 73.36 million tons of production in 2020 (FAOSTAT, 2020). In the past ten years, cucumber angular leaf spot (ALS) disease, which is caused by *Pseudomonas amygdali* pv. *Lachrymans* (*Pal*), has become one of the most important causes of cucumber loss worldwide (Bhat et al., 2010; Gomila et al., 2017; Arnold and Preston, 2019; Peix et al., 2018; Li et al., 2019). In addition, *Pal* can attack a wide range of cucurbits, including *Citrullus lanatus* (watermelon), *C. melo* (muskmelon), *Cucurbita maxima* (squash) and *C. pepo* (pumpkin) (Olczak-Woltman et al., 2009; Bhat et al., 2010; Li et al., 2019). The traditional route of transmission of the globally distributed *Pal* has been considered to be soil-borne, seed-borne, wind, rain and insect-borne transmission (Leben, 1981; Umekawa et al., 1981; Groth et al., 1995; Hirano et al., 1996; Hansen, 2009). Recently, we showed that *Pal* aerosols represent an effective mechanism for the spread of ALS disease in greenhouses, and cucumber plant canopies at the early stage of disease development serve as the main source of *Pal* aerosol (Chai et al., 2020).

Bioaerosols may play an important role in the transmission of pathogens to plants, which can lead to epidemic outbreaks (Allegra et al., 2016; Joung et al., 2017). Bioaerosol can be generated by overhead irrigation in greenhouses, raindrops hitting soil and mechanical crushing of diseased plants in fields (Quinn et al., 1980; Joung et al., 2017). The transmission of pathogenic bacteria by aerosols is potentially influenced by many factors, including the concentration and particle size distribution of bacteria-laden particles, how long airborne bacteria remain infectious under different environmental conditions, which can affect the concentration and viability.

Environmental conditions, including temperature, relative humidity (RH), UV radiation, and air flow, profoundly affect the survival of airborne microorganisms, among which temperature and RH are the major factors affecting bacterial survival in aerosol (Webb, 1961; Moe and Harper, 1983; Ariya and Amyot, 2004; Thompson et al., 2011; Hoeksma et al., 2015; Afsharnia et al., 2018; Islam et al., 2020). Temperature directly affects the metabolism and reproduction of the bacteria, and RH affects the concentration of the ambient bacteria, and these findings are dependent on species and metabolic factors (Burrows et al., 2009; Thompson et al., 2011). For example, the viability of *Escherichia coli* in aerosols decreases with the decline in RH, and the death rates of *E. coli* at 30°C were approximately four times faster than those at 15°C at the same RH (Wathes et al., 1986). Various species of bacteria have shown distinctly different adaptations to different levels of temperature and RH. RH have been reported to have lethal effects on the survival of some species in air, e.g., *E. coli* (Wathes et al., 1986), *Legionella pneumophila* (Hambleton et al., 1983) and *Yersinia pestis* (Gut et al., 2016); while the survival of *Staphylococcus epidermidis* (Thompson et al., 2011) was demonstrated to not be affected by RH. It was reported that the survival of *P. syringae* in aerosols was greatest at 70%-80% RH and 12°C in a field environment (Marthi et al., 1990). However, little is known about the influence of environmental parameters on the survival of *Pal* aerosols in greenhouses.

Temperature and RH play important roles in the emission and transmission of bioaerosols as well as in bacterial disease outbreaks (Harrison, 1980; Xin et al., 2016; Huot et al., 2017; Kowalski and Pastuszka, 2018). Plant canopies are considered a source of airborne bacteria (Lindemann et al., 1982; Lindemann and Upper, 1985; Mcinnes et al., 1988). The plant phyllosphere provides an important habitat for microorganisms to colonize and proliferate (Melotto et al., 2008; Zeng et al., 2010; Xin et al., 2016). Some studies have shown that the populations of *P. syringae* on the leaf surface increased by orders of magnitude after heavy rain, which caused an increasing number of bacterial cells to be transferred to the atmosphere (Hirano and Christen, 2000; Bigg et al., 2015). The dispersal of bacterial aerosols and deposition on healthy plants will accelerate an epidemic of bacterial disease. In addition, phyllosphere bacterial disease outbreaks usually require a period of high RH (Aung et al., 2018), and a suitable temperature promotes the virulence of bacterial pathogens and the interaction between plants and pathogens (Xin et al., 2016; Aguilera et al., 2017). However, studies have not been undertaken to investigate the dynamics of airborne *Pal* present in cucumber greenhouses and their dependence on temperature and RH.

The size distribution of bioaerosols varies systematically with air temperature and RH, which could affect the behavior of bioaerosols in air. The particle size of bioaerosols has been shown to increase by hygroscopic growth, e.g., coronavirus aerosols presented a larger diameter at 25°C and 79% RH than at 38°C and 24% RH (Liao and Luo, 2005; Pyankov et al., 2018). The particle size as well as the motion of aerosols, such as resuspension, transport by air movement and sedimentation, have been shown to influence the possibility of microbes entering the atmosphere (Liao and Luo, 2005). For example, dust of 30 and 100 μm is most likely to be raised from the surface to the atmosphere (Chamberlain, 1975). In addition, the survivability of a biological particle in the atmosphere will also be affected by its size (Jones and Harrison, 2004). The concentrations of *E. coli* in PM10, PM2.5 and PM1 are higher in wet aerosols than in dry aerosols (Hoeksma et al., 2015).

Previous studies have shown that the effects of temperature and RH on the release and survival of bacterial aerosols are distinct based on the various species of bacteria. However, little is known about the influence of temperature and RH on the survival, release and pathogenicity of *Pal* aerosols. In the present study, we extend the findings of our previous study to (i) assess the survival the size distribution of *Pal* aerosols under different temperature and RH levels; (ii) evaluate the release of *Pal* aerosols from diseased cucumber in aerosol chambers and greenhouse environments under different temperature and RH levels; and (iii) investigate the infectivity of *Pal* aerosols to cucumber seedlings at different temperature and RH levels. These data will provide a foundation for the effective alleviation and control of ALS disease by controlling temperature and RH.

## 2 Materials and methods

### 2.1 Bacterial strain, culture conditions and inoculum preparation

A green fluorescence protein gene (GFP)-marked *Pal* strain (*Pal::*GFP), which showed consistent fluorescence and was indistinguishable from the wild-type strain in growth rate and pathogenicity, was used in this study (Chai et al., 2020).

The *Pal*::GFP strain was grown at 28°C for 48 h on nutrient agar (NA) plates (10.0 g of peptone, 3.0 g of beef powder, 5.0 g of NaCl and 15.0 g of agar per liter; pH 7.0; Solarbio Science & Technology Co., Ltd., Beijing, China) containing 50 μg/mL kanamycin (referred to as NK). Then, single colonies were selected and cultured in nutrient broth (NB) medium (containing 10.0 g of peptone, 3.0 g of beef powder and 5.0 g of NaCl per liter; pH 7.0) supplemented with 50 μg/mL kanamycin for 24 h at 28°C with shaking at 200 rpm. The concentration of the *Pal*::GFP suspension was determined by the optical density at 600 nm (OD_600_ = 0.8, approximately 1 × 10^8^ CFU/mL).

### 2.2 Cucumber variety

The experimental cucumber variety ‘Zhongnong no. 6’ (China Vegetable Seed Technology Co., Ltd., Beijing), a typical Huabei-type cucumber of northern China, was used in this study. The healthy cucumber seeds used in this study were first confirmed to be free of *Pal* by the traditional agar planting method and PCR using the primer pair Pslgap1-F/R as described by Meng et al., 2016.

### 2.3 Environmentally controlled aerosol chamber

Environmentally controlled aerosol chambers comprising organic glass with a size of 70 cm × 60 cm × 60 cm (length × width × height) were used in this study (**Figure S1**). The air temperature and RH in the aerosol chamber were controlled by an air conditioner (Haier, KFRd-27N/PAA12, China) and an air humidifier (Yadu, SC-EB35B, China), respectively, to maintain constant temperature and RH conditions (Chai et al., 2020). The collision nebulizer (BGI, Inc., Waltham, MA) and the Andersen sampler (Thermo-Andersen, Smyrna, GA, USA) were connected to the air inlet and outlet, which were located on the left and right sidewalls of the aerosol chamber, respectively. A fan and a UV light were installed at the top wall for mixing the *Pal* aerosol well inside the chamber and sterilization, respectively. The door was fixed at the front wall with six screws, and a silicone pad was applied to ensure that the chamber was airtight.

### 2.4 Effect of temperature and RH on the release of *Pal* aerosol from diseased cucumber seedlings

**Figure 1:**
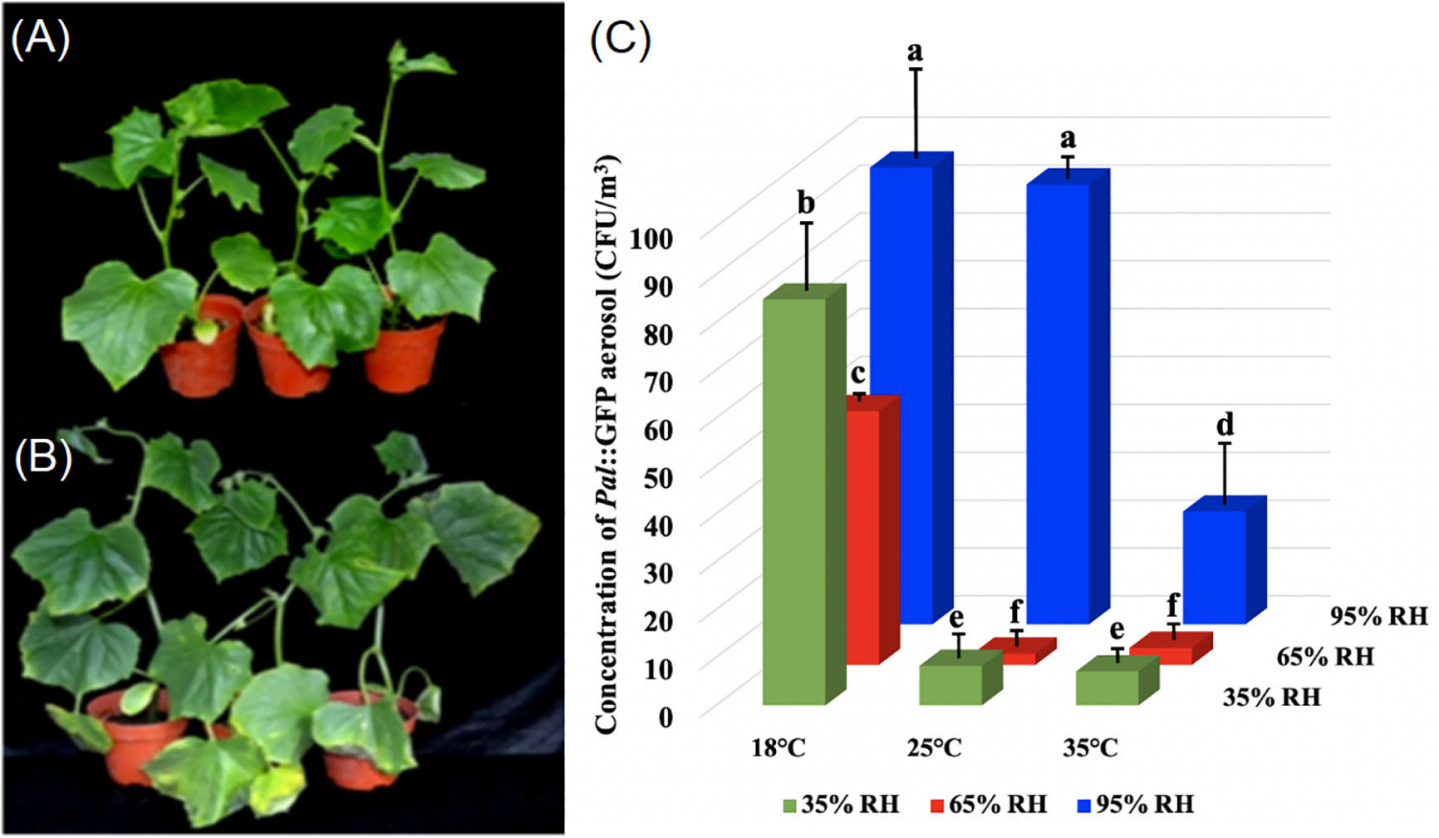
Effect of temperature and relative humidity (RH) on the release of *Pseudomonas amygdali* pv. *lachrymans* (*Pal*) in aerosols from infected cucumber plants. Twenty cucumber plants at the early stage of the angular leaf spot (ALS) disease, were placed in nine exposure chambers, which maintained at temperatures of 18, 25 and 35°C with humidity levels of 35%, 65% and 95% RH per temperature, respectively. **(A)**, Cucumber plants inoculated with sterile water. **(B)**, Symptoms on cucumber plants at the early stage of the ALS disease, which inoculated with *Pal*::GFP suspension. **(C)**, The release amount of *Pal*::GFP in aerosols, which detected at 24 h after incubation under different conditions of temperature and RH levels. The experiments were independently repeated three times. Columns and error bars represent the mean values and standard deviations (SDs) from three replicates. Columns labeled with different letters indicate statistically significant differences (*p*< 0.05).

Twenty cucumber seedlings with two true leaves were inoculated with a 10^8^ CFU/mL suspension of *Pal*::GFP by spraying and incubated at 90% RH and 25°C for 48 h for the development of ALS disease (**Figures 1A, B**). Then, the infected cucumber seedlings at the early stage of ALS disease were transferred to nine aerosol chambers, which were maintained at three temperatures (18, 25 and 35°C) with three humidity levels (35%, 65% and 95% RH) per temperature. A total of nine temperature-humidity combinations were tested. The release of *Pal*::GFP aerosol from infected cucumber seedlings was detected at 24 h after incubation at different levels of temperature and RH. The Andersen sampler (Thermo-Andersen, Smyrna, GA, USA) was used to collect *Pal*::GFP aerosol with a flow of 28.3 L/min for 10 min. For each treatment, the bioaerosol samples were collected onto 90 mm diameter NK plates and then cultured at 28°C for 48 h, and the *Pal*::GFP colony-forming units (CFU) were counted (**Figure S2**). The experiments were independently repeated three times.

### 2.5 Effect of temperature and RH on the survival dynamics and size distribution of *Pal* in aerosols

A 10^8^ CFU/mL suspension of *Pal*::GFP was aerosolized by a six-jet Collison nebulizer (BGI, Inc., USA), at a flow rate of 12 L/min for 30 min, to nine aerosol chambers, in which different levels of temperature (18, 25 and 35°C) and RH (35%, 65% and 95% RH) were maintained. Aerosol samples were collected at 0, 5, 15, 30, 45, 60 and 75 min after aerosolization. A six-stage Andersen sampler (Andersen Inc, USA) with a flow rate of 28.3 L/min was used to collect the aerosol samples onto 90 mm diameter NK plates, which were then incubated at 28°C for 48 h, and the *Pal*::GFP CFUs were counted to evaluate the initial concentration, survival dynamics and particle size distribution of *Pal* aerosols. The experiments were repeated three times.

The initial concentration of *Pal*::GFP aerosol at different levels of temperature and RH was determined with aerosol samples collected at 0 min after nebulization. The size distribution of *Pal*::GFP aerosols at different levels of temperature and RH was assessed by an Andersen sampler based on their inertia-related aerodynamic diameter (Xu et al., 2013). The survival dynamics and decay rate of *Pal*::GFP in aerosols at different levels of temperature and RH were calculated using the first-order decay model according to Eq. (1) (Clifton et al., 2010). In this model, the decay rate k represents the combined effect of natural biological decay arising from the loss of bacterial viability and the effect of particle deposition on the chamber surfaces. The half-life was calculated according to Eq. (2), and the value of k was obtained from Eq. (1), which was determined by the *Pal*::GFP aerosol decay experiment. Survival curves for *Pal*::GFP in aerosols generated in aerosol chambers under different environmental conditions were obtained using GraphPad Prism 7.0 (GraphPad Software, Inc.).


(1) $${\text{Log}}_{\text{e}}\left({\text{N}}_{t}\right)\;=\;{\text{log}}_{\text{e}}\left({\text{N}}_{0}\right)\;-\text{ kt}$$

(2) $${\text{t}}_{1/2}=\frac{{\text{log}}_{\text{e}}\text{(0}.5)}{\text{k}}$$

$${\text{N}}_{0}=\text{ concentration of bacteria at time }0\;\left(\text{CFU}/{\text{m}}^{3}\right)$$

$${\text{N}}_{\text{t}}=\text{ concentration of bacteria at time t }\left(\text{CFU}/{\text{m}}^{3}\right)$$

$$\text{k}=\text{decay rate }\left(\text{mi}{\text{n}}^{-1}\right)$$

t=time (min)

$${\text{t}}_{1/2}=\text{half}-\text{life }\left(\text{min}\right)$$

### 2.6 Effect of temperature and RH on the infectivity of *Pal* in aerosols and colonization on cucumber leaves

The effects of temperature and RH on the infectivity of *Pal*::GFP aerosol to cucumber seedlings were examined in nine independent aerosol chambers (**Figure S1**) in which twenty healthy cucumber seedlings were placed in advance, and the chambers were maintained at temperatures of 18, 25 and 35°C with humidity levels of 35%, 65% and 95% RH. A *Pal*::GFP suspension (10^8^ CFU/mL) was used to generate aerosol by the six-jet Collison nebulizer at a flow rate of 12 L/min for 2 h. The mixing fan was run at an average velocity of 0.4 m/s during the period of nebulization. The disease index (DI) of cucumber ALS disease were examined 7 days after exposure to *Pal*::GFP aerosol according to the method described previously (Meng et al., 2016).

The populations of *Pal*::GFP colonized on symptomatic cucumber leaves were also determined. Briefly, three infected leaves were sampled from each cucumber plant, and four leaf discs (1.0 cm in diameter) were taken using a cork borer. The leaf discs were surface sterilized for 1 min in 75% ethanol, rinsed three times in sterile water (Xin et al., 2016), homogenized in 1 mL sterile water using a mortar and serially diluted and plated on NK plates to determine the CFU (**Figure S2**). After incubation at 28°C for 48 h, the number of colonies was counted and normalized to the value per cm^2^ of leaves (Rajendran et al., 2016). The experiments were independently repeated three times.

### 2.7 Effect of temperature and RH on the concentration of *Pal* aerosol in naturally infested cucumber greenhouses

To determine the effect of temperature and RH on the concentration of *Pal* aerosol and disease severity in cucumber greenhouses, aerosol samples were collected from nine greenhouses where cucumber ALS disease occurred naturally from 2019 to 2021. The infested greenhouses were located in Wuqing district, Tianjin city (greenhouses I and II); Guantao county, Hebei province (greenhouses III, IV, and V); and Shouguang county, Shandong province (greenhouses VI, VII, VIII, and IX).

A portable sampler (HighBioTrap, Beijing Blue Tech, Inc., Beijing, China) was used to sample aerosol at 1.5 m above the ground in the middle of the greenhouses. The sampler was operated at a flow rate of 1000 L/min, with an impact velocity of approximately 10.2 m/s (S/W = 1.5, T/W = 1) and a cutoff size of ~ 2 μm (Chen and Yao, 2018). For each sampling greenhouse, bioaerosols were collected onto 90 mm (diameter) sterilized aluminum membranes coated with 600 μL of mineral oil. Three independent aerosol samples were collected from 9:00 am to 10:00 am daily for three continuous days and served as three biological replicates. A total of 27 air samples were collected from nine greenhouses. Then, the aerosols were assessed by quantitative PCR (qPCR). Briefly, the mineral oil membrane with aerosols was immediately transferred to a 50 mL centrifuge tube and washed using 3 mL of Tween 20 suspension. Then, the obtained oil-in-water emulsion was centrifuged at 8000 rpm to remove the mineral oil. The pellet remaining in the centrifuge tube was then used for genomic DNA extraction with a TIANamp Bacteria DNA Kit (Tiangen Biotech, Inc., Beijing, China) according to the manufacturer’s guidelines. The concentration of *Pal* in aerosols was detected by qPCR with the primer pair Pslgap1-F/R according to the protocol described in our previous work (Meng et al., 2016; Chai et al., 2020). The DIs of cucumber ALS disease were also recorded. The temperature and humidity data in greenhouses were obtained using a digital thermohygrometer (DL-WS 210, Gsome Technology Co., Ltd, Hangzhou, China).

Due to the nonnormal distribution of variables describing the concentration of *Pal* in aerosols under various temperature and RH environments, a nonlinear regression model was assumed and constructed using Origin software (OriginLab, version 8.0). A 3D surface plot (Kowalski and Pastuszka, 2018) was used to determine and visualize the influence of temperature and RH on the concentration levels of *Pal* in aerosols.

### 2.8 Statistical analysis

One-way ANOVA and Duncan’s test were used for statistical analysis by SPSS 22.0 software, and p values < 0.05 indicated a statistically significant difference.

## 3 Result

### 3.1 Effect of temperature and RH on the aerosol release of *Pal* from diseased cucumber seedlings

Cucumber seedlings at the early stage of ASL disease (**Figure 1B**) were incubated for 24 h in nine aerosol chambers, which were maintained at temperatures of 18, 25 and 35°C with humidity levels of 35%, 65% and 95% RH, and the concentrations of *Pal*::GFP aerosol were examined. The results demonstrate that temperature and RH are both significant factors influencing the release of *Pal*::GFP aerosols from diseased cucumber. High humidity (95% RH) at comparatively low temperature (18°C) is conducive to the release of *Pal*::GFP aerosols. The highest release amount of *Pal*::GFP aerosol (96 CFU/m^3^) from diseased cucumber was found at 18°C and 95% RH, while the lowest concentrations of *Pal*::GFP aerosol (<10 CFU/m^3^) were detected at relatively high temperature (25°C and 35°C) and low humidity (35% RH and 65% RH). Under dry conditions (≤ 65% RH), the amount of *Pal*::GFP aerosols released from cucumber seedlings was significantly lower than that under high RH conditions (95% RH). However, the lower amount of *Pal*::GFP aerosols released under dry conditions (≤ 65% RH) could be improved by reducing the temperature from high temperature (25°C and 35°C) to low temperature (18°C), with *Pal*::GFP aerosol concentrations increasing from< 10 CFU/m^3^ to 84 CFU/m^3^ (**Figure 1C**).

### 3.2 Effect of temperature and RH on the survival dynamics of *Pal* in aerosols

**Figure 2:**
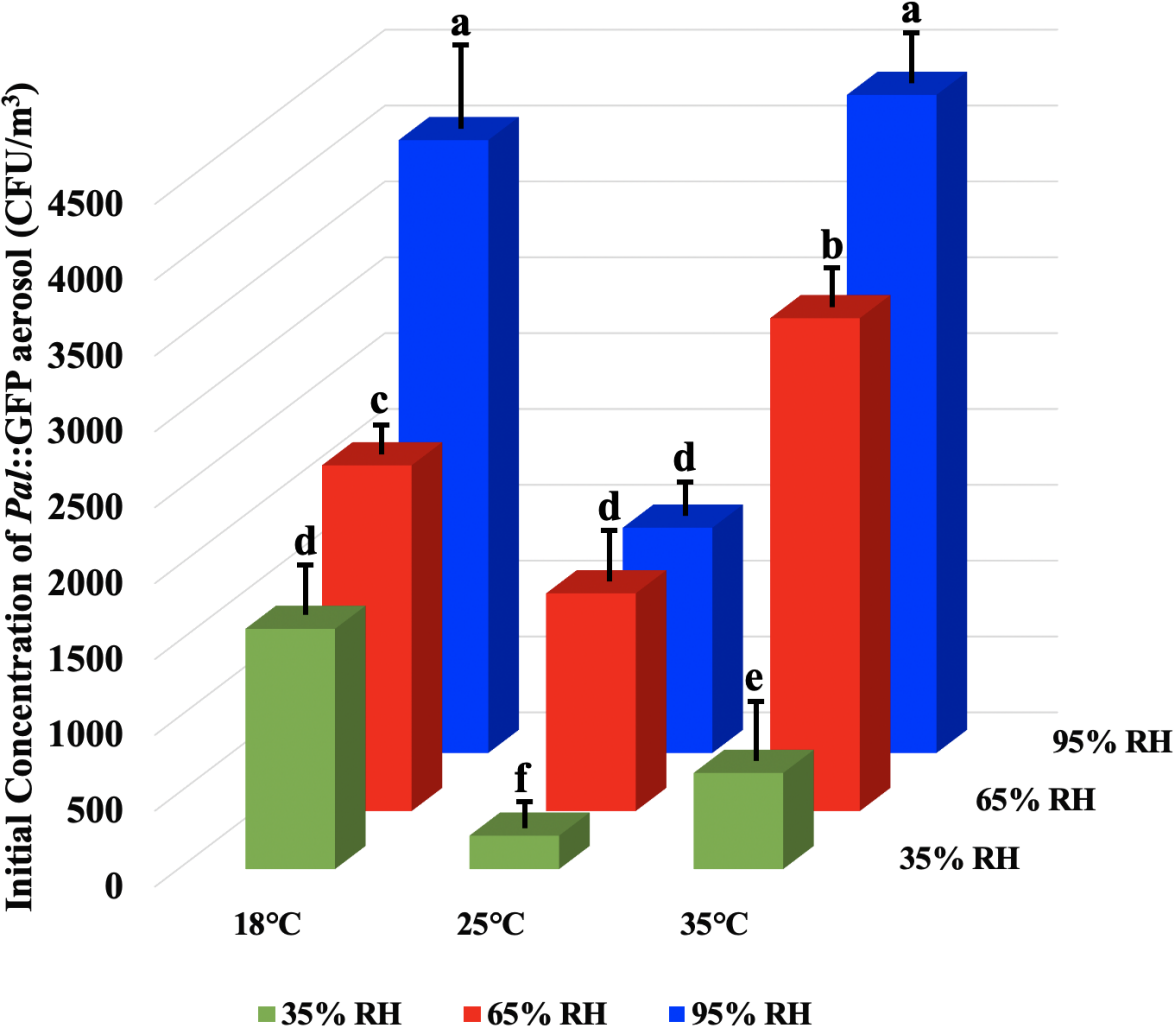
Effect of temperature and relative humidity (RH) on the initial concentration of *Pseudomonas amygdali* pv. *lachrymans* (*Pal*) in aerosols after aerosolization. *Pal*::GFP suspension (10^8^ CFU/mL) was aerosolized to nine aerosol chambers, which maintained at 18, 25 and 35°C with three humidity levels of 35%, 65% and 95% RH per temperature, respectively, and the initial concentrations of *Pal*::GFP in aerosols were examined 0 min after nebulization. The experiments were independently repeated three times. Columns and error bars represent the mean values and standard deviations (SDs) of three replicates. Columns labeled with different letters indicate statistically significant differences (*p*< 0.05).

*Pal*::GFP was aerosolized to nine aerosol chambers, which were maintained at 18, 25 and 35°C with three humidity levels of 35%, 65% and 95% RH, and *Pal*::GFP concentrations were examined from 0 to 75 min. The initial concentrations of *Pal*::GFP in aerosols, which were examined 0 min after nebulization, were largely affected by RH, showing a downtrend with the decrease in humidity levels at the same temperature (**Figure 2**). The highest initial concentrations of *Pal*::GFP in aerosols were detected at 95% RH, with 4040 ± 590 CFU/m^3^ at 18°C and 4338 ± 260 CFU/m^3^ at 35°C. The lowest initial concentrations of *Pal*::GFP aerosol were detected at 35% RH, with 1584 ± 291 CFU/m^3^ at 18°C and 634 ± 305 CFU/m^3^ at 35°C. Inexplicably, the initial concentrations of *Pal*::GFP in aerosols generated at 25°C were always lower than those in aerosols generated at 18°C and 35°C at the same RH levels.

**Figure 3:**
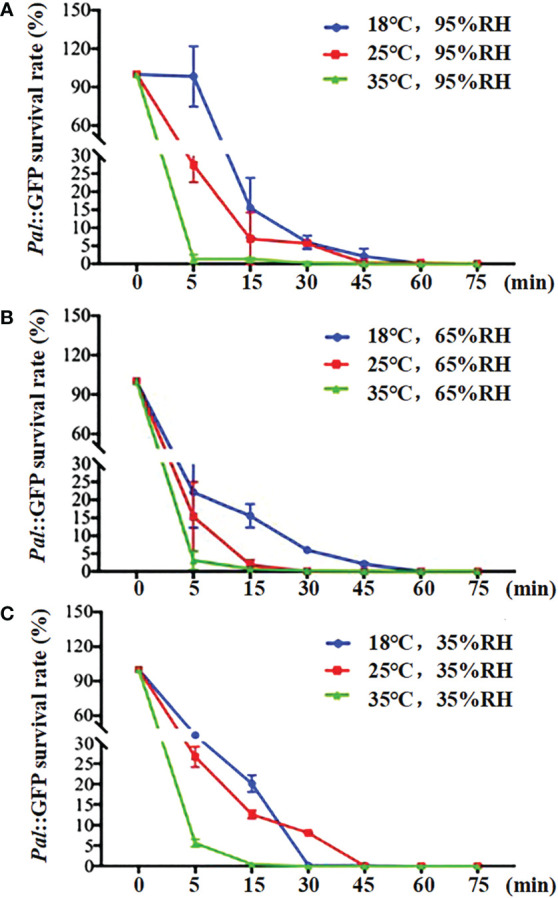
Effect of temperature and relative humidity (RH) on the survival rate of *Pseudomonas amygdali* pv. *lachrymans* (*Pal*) in the aerosol chambers. **(A)** 95% RH, **(B)** 65% RH and **(C)** 35% RH. The survival rates of *Pal*::GFP aerosol were determined at 0, 5, 15, 30, 45, 60 and 75 min after aerosolization, respectively. Error bars represent the standard deviations (SDs) of three replicates.

Generally, the decay rate of *Pal*::GFP in aerosols showed a decreasing trend with rising temperature and falling RH (**Figure 3**). *Pal*::GFP in aerosols decayed exponentially with a calculated decay rate of 0.0881-0.1192 min^-1^, corresponding to a half-life of 1.98-6.79 min (**Table 1**). The fastest decay rate (0.0881 min^-1^) of *Pal*::GFP in aerosols was detected at 35°C and 35% RH, corresponding to a half-life of 1.98 min (**Table 1**, **Figure 3C**). The slowest decay rate of *Pal*::GFP in aerosols was detected at 95% RH, corresponding to half-lives of 6.27 min at 35°C and 6.79 min at 25°C, respectively (**Table 1**, **Figure 3A**).

**Table 1:** Effect of temperature and relative humidity (RH) on *Pseudomonas amygdali* pv. *lachrymans* (*Pal*) survival in aerosols.

Relative humidity	Temperature	Decay rate (k) (min^-1^)	Half-life (t_1/2_) (min)	R^2^ values	Survival time (min)
	18°C	0.1169	5.82	0.9507	75
95% RH	25°C	0.1024	6.79	0.9579	60
	35°C	0.1104	6.27	0.7994	60
	18°C	0.1168	5.93	0.7397	60
65% RH	25°C	0.1192	5.81	0.9580	45
	35°C	0.1181	5.86	0.9198	45
	18°C	0.1324	5.23	0.9081	60
35% RH	25°C	0.0601	3.56	0.8714	45
	35°C	0.0881	1.98	0.9497	30

Pal aerosol were generated by a six-jet Collison nebulizer at the flow rate of 12 L/min for 30 min in exposure chambers maintained at temperatures of 18, 25 and 35°C with humidity levels of 35%, 65% and 95% RH. Aerosol samples were collected at 0, 5, 15, 30, 45, 60 and 75 min after aerosolization. The decay rate and half-life of Pal aerosol were calculated according to first order decay model. R^2^ values mean the correlation coefficient in decay models.

The survival rate of *Pal*::GFP in aerosols decreased significantly during the first 15 min after aerosolization, and few *Pal*::GFP cells remained at 75 min after aerosolization. Temperature was found to be a crucial factor that influenced the survival dynamics of *Pal* in aerosols. A significant decrease (*p* < 0.05) in the *Pal* survival rate was observed as the temperature increased from 18°C to 35°C at the same RH levels. At 95% RH conditions, a larger decay was observed within 5 min after aerosolization at 35°C, with a survival rate of 1.33%, which was significantly lower (*p* < 0.05) than that at 18°C (98.41%) and 25°C (27.42%). A similar trend was also obtained at 65% RH and 35% RH; as the temperature increased from 18°C to 35°C at 5 min, the survival rate decreased from 22.88% to 3.15% and 35.04% to 5.46%, respectively (**Figure 3**). In addition, the decay rate of *Pal*::GFP increased as the RH decreased.

### 3.3 Effect of temperature and RH on the particle size distribution of *Pal* aerosols

**Figure 4:**
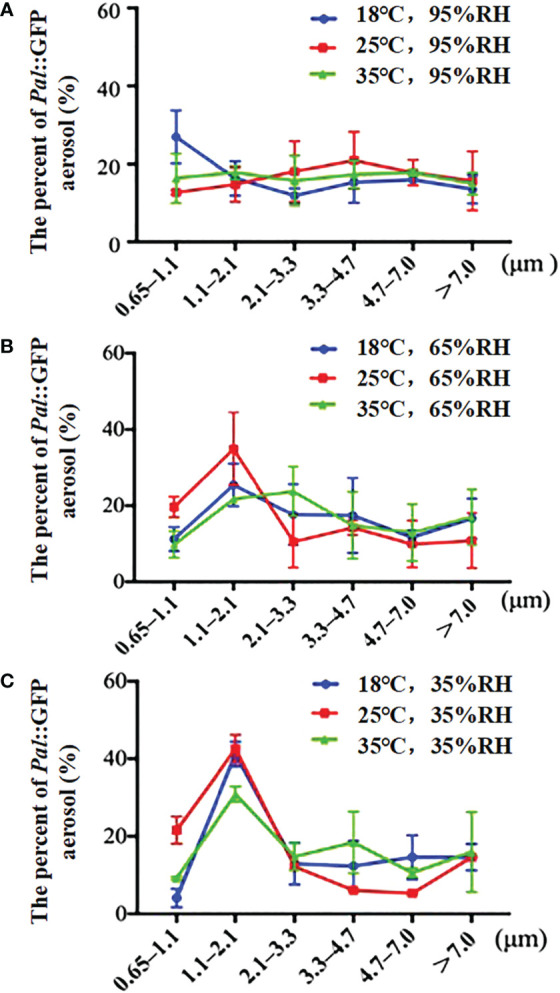
Effect of temperature and relative humidity (RH) on the size distribution of *Pseudomonas amygdali* pv. *lachrymans* (*Pal*) particles in the aerosol chambers. The size distribution of *Pal*::GFP aerosol were detected immediately after aerosolizing *Pal*::GFP suspension (10^8^ CFU/mL) for 30 min at the flowrate of 12 L/min by a six -jet Collision nebulizer. Error bars represent the standard deviations (SDs) of three replicates.

The size distributions of *Pal*::GFP aerosols under different temperature (18, 25 and 35°C) and humidity (35%, 65% and 95% RH) conditions were analyzed according to the inertia-related aerodynamic diameter of the six-stage Andersen sampler. Generally, RH played a critical role in the particle size distribution. *Pal*::GFP aerosols presented a smaller diameter under low humidity than under high humidity conditions. The proportions of *Pal*::GFP aerosols detected in stage 5 (1.1-2.1 μm) at 35% RH (30.87%-42.59%) were significantly higher than those at 65% RH (21.72%-34.85%) and 95% RH (14.72%-7.82%) (**Figure 4**). However, no significant differences were observed in the size distribution of *Pal*::GFP aerosols among different levels of temperature.

### 3.4 Effect of temperature and RH on the infectivity and colonization of *Pal* in aerosols

**Figure 5:**
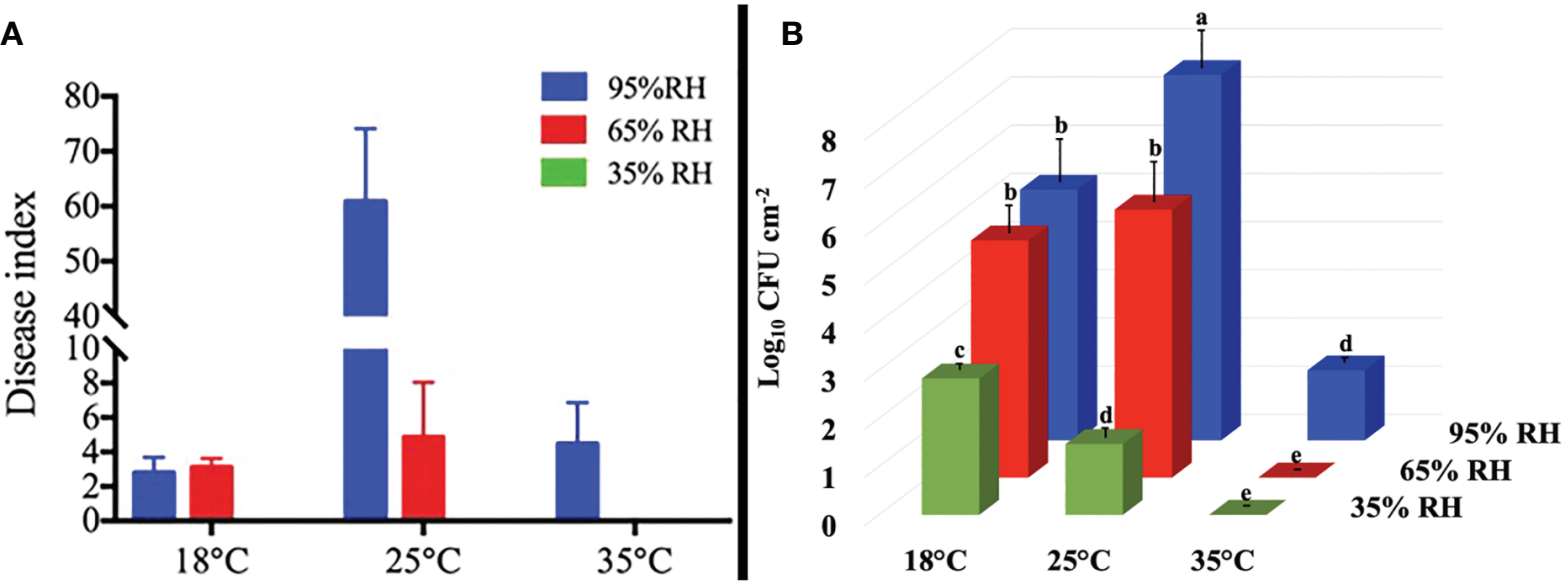
Effect of temperature and relative humidity (RH) on the pathogenicity and colonization of *Pseudomonas amygdali* pv. *lachrymans (Pal*) in aerosols on cucumber leaves. Healthy cucumber plant were exposed to *Pal*::GFP aerosol, generated by a six-jet Collison nebulizer for 30 min at the flow rate of 12 L/min, in chambers at different levels of temperature and RH. **(A)** The disease index and **(B)** colonization of *Pal*::GFP in cucumbers leaves were measured at 7 days after exposed to *Pal*::GFP aerosols at different levels of temperature and RH. The experiments were independently repeated three times. Columns and error bars represent the mean values and standard deviations (SDs) of three replicates. Columns labeled with different letters indicate statistically significant differences (*p*< 0.05).

The symptoms and severity of cucumber ALS disease caused by *Pal* aerosol were influenced by physiological or environmental factors, especially temperature and RH. The optimum conditions for cucumber ALS disease development were 25°C and 95% RH, with a DI of 60.9; however, the disease developed slightly at 18°C and 35°C, with DI values lower than 10.0. Meanwhile, the DI decreased with the decline in RH. At 25°C, when cucumber seedlings were exposed to *Pal*::GFP aerosols at 65% RH, the DI decreased to 4.86. No visible symptoms were observed when cucumber was exposed to *Pal*::GFP aerosols at 35% RH (**Figure 5A**).

In addition, the populations of *Pal*::GFP colonized on cucumber leaves were also evaluated. Concomitantly, the highest level of *Pal*::GFP multiplication was observed at 25°C and 95% RH, with *Pal*::GFP concentrations of 10^7.58^ CFU/cm^2^ (**Figure 5B**). The population of *Pal*::GFP decreased with increasing temperature and decreasing RH, and low *Pal*::GFP concentrations were detected on cucumber leaves at 35°C and RH ≤ 65% (**Figure 5B**).

### 3.5 Effect of temperature and RH on the concentration of airborne *Pal* in the naturally infested cucumber greenhouse

The concentration of *Pal* cells was specifically detected in bioaerosol samples collected from naturally infested greenhouses by qPCR (**Table S1**). The highest levels of *Pal* cells (825-955 cells/m^3^) were found in aerosol samples collected from greenhouses I, V and VI located in Tianjin city, Hebei and Shandong provinces, with temperatures of 19.0-23.1°C and RHs of 91.5%-95.1%. Relatively high levels of *Pal* cells (173-215 cells/m^3^) were obtained from greenhouse IV located in Hebei province, with temperatures of 16.7-18.8°C, and RHs of 69.8%-75.9%. The lowest levels of *Pal* cells (27-61 cells/m^3^) were obtained from greenhouses II and IX located in Tianjin city and Shandong province, with temperatures of 29.3-31.4°C and RHs of 30.2%-40.9%.

**Figure 6:**
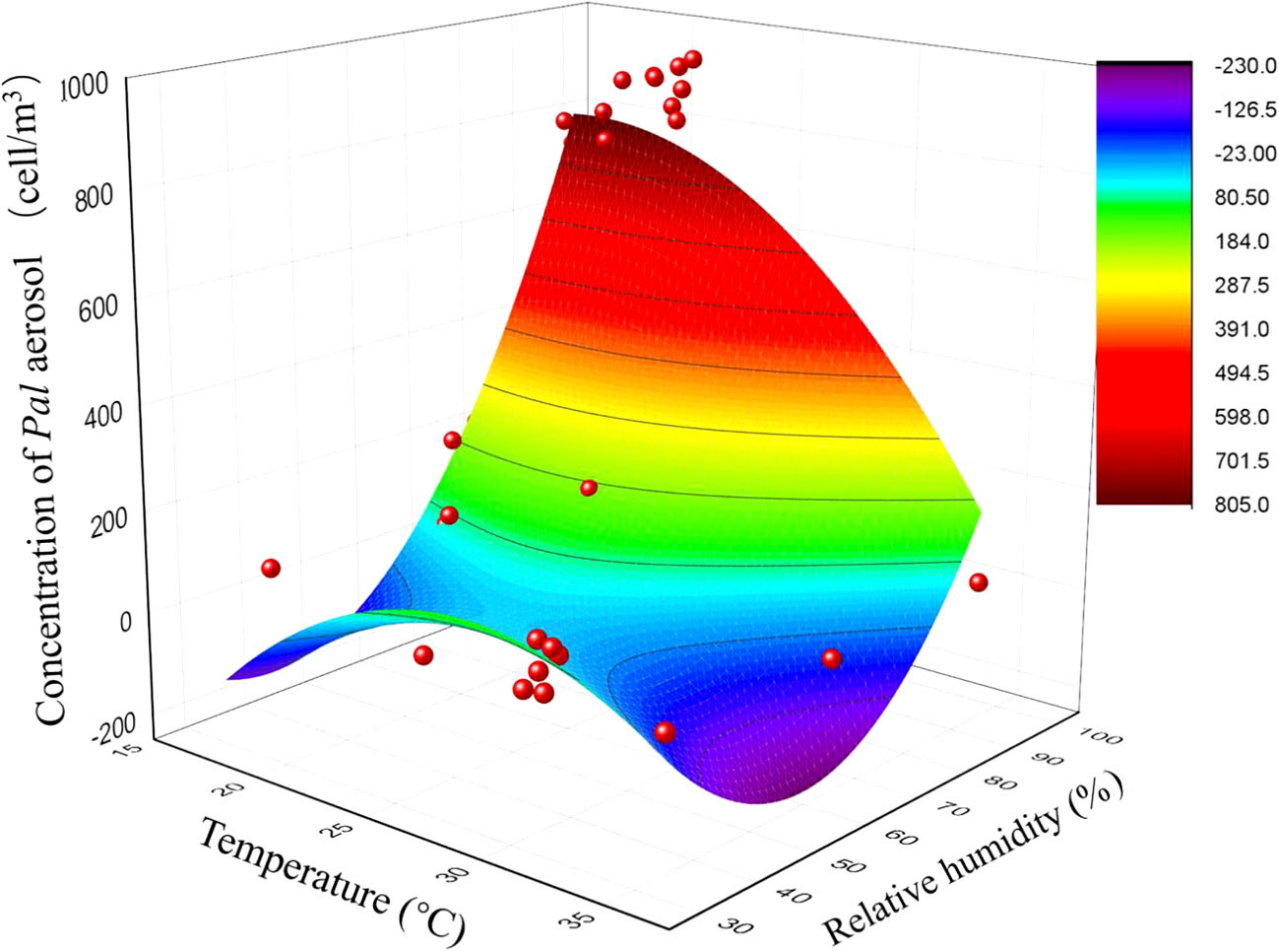
The 3D surface plot of the co-influence of temperature and relative humidity (RH) on concentration levels of *Pseudomonas amygdali* pv. *lachrymans* (*Pal*) in aerosols in naturally infested cucumber greenhouses. Bioaerosol samples were collected from nine naturally infested greenhouses, which located in Tianjin City (n=2), Hebei (n=3) and Shandong (n=4) Provinces, from 2019 to 2021. The concentration of *Pal* in aerosols was detected by qPCR. Temperature and RH in greenhouses were obtained using a digital thermo-hygrometer. The co-influence of temperature and RH on *Pal* aerosol concentration presented as a six-parameter model: Z = Z_0_ + ax + by + cxy + dx^2^ + fy^2^. In which, Z = *Pal* aerosols concentration, x = temperature, y = RH.

The surface response of the concentration levels of *Pal* aerosol to the combined effects of temperature and RH was obtained by the equation Z = Z_0_ + ax + by + cxy + dx^2^ + fy^2^ (**Table 2**). A lower temperature and high RH caused a larger amount of *Pal* release in greenhouses, and the most suitable conditions for *Pal* aerosol release were approximately 22°C-23°C and 100% RH (**Figure 6**).

**Table 2:** Parameter estimates of the non-liner regression model describing the co-influence of temperature and relative humidity (RH) on the concentration levels of *Pseudomonas amygdali* pv. *lachrymans* (*Pal*) in aerosols.

Parameter	Estimate	Standard error
Z_0_	– 1425.03	1278.35
a	140.01	71.76
b	– 12.81	19.37
c	– 0.68	0.35
d	– 2.13	1.28
f	0.31	0.11

The model is a six-parameter model: Z = Z_0_ + ax + by + cxy + dx^2^ + fy^2^. In the model, Z = the concentration of Pal aerosols, x = T, y = RH, Z_0_, a, b, c, d and f are parameters. In the non-liner regression model, reduced Chi-Sq is 48171.68, R-Square (COD) is 0.71, and Adj. R-Square is 0.67.

## 4 Discussion

Plant canopies are considered a source of airborne bacteria, and the amount of bacterial aerosol released is different under various environments (Lindemann et al., 1982; Lindemann and Upper, 1985; Mcinnes et al., 1988). Bacteria on plant surfaces can generally be regarded as being inertly released into the atmosphere due to raindrops or shaking (Robertson and Alexander, 1994; Jones and Harrison, 2004). However, our previous study suggested that *Pal* aerosols could be emitted to the atmosphere in cases without rain or other external forces. Such emissions of *Pal* aerosol to the atmosphere may play a critical role in the dispersal of cucumber ALS disease in greenhouses (Chai et al., 2020). To estimate the potential extent of *Pal* aerosol emissions under different temperature and RH, we performed experiments both in aerosol chambers and in infested cucumber greenhouses located in different areas. Our experimental measurements revealed that during the release of *Pal* aerosol from infected cucumber plants, *Pal* in aerosols can reach the highest concentrations under conditions of 95% RH at a low temperature (≤ 25°C), with approximately 100 CFU/m^3^ in the aerosol chamber detected by an Andersen sampler (**Figure 1C**) and 1000 cells/m^3^ in the greenhouse detected by a portable sampler (**Figure 5B**). The concentration of *Pal* in aerosols under high temperature (35°C) and low RH (35% RH) was less than 50 CFU/m^3^. In addition, the decay rate of *Pal* in aerosols also accelerated (0.0881 min^-1^) under high temperature and low RH. Taken together, the inability of *Pal* in aerosols to cause cucumber ALS disease under high temperature and low RH could be explained by the release amount of *Pal* in aerosols being lower than the infection threshold value of *Pal* in aerosols (84-179 CFU/m^3^), the shorter survival time and the more rapid decay rate (Chai et al., 2020). Previous studies have demonstrated that the optimal conditions for the development of cucumber ALS disease were 25°C and more than 90% RH (Umekawa and Watanabe, 1980; Umekawa and Watanabe, 1982).

The initial concentrations of airborne bacteria during aerosolization and airborne suspension have been shown to be significantly different at various temperature and RH levels (Clifton et al., 2010; Ng et al., 2018). The initial concentration and survival rate of *E. coli* in aerosols at high RHs of 60%-80% was shown to be much higher than those at lower RH of 40% (Ng et al., 2018). In this study, the initial concentrations of *Pal*::GFP after aerosolization showed a downward trend with the decrease in RH levels at the same temperature, which was similar to the results for *E. coli* in aerosols. It has been suggested that the different damage levels of membrane integrity and fluidity index (FI) of *Pal* in aerosols are produced during aerosolization under different temperatures and RHs, which may lead to the difference in the initial concentration of *Pal*::GFP in aerosols (Ng et al., 2018).

The effect of temperature and RH on the survival of bacteria in aerosols varies based on the bacterial species. For example, the decay rate of *E. coli* in aerosols was significantly greater at low RH (<50% RH) at 15°C or 30°C than at high RH (Wathes et al., 1986). The death rate of *Pasteurella tularensis* in aerosols increased with increasing environmental temperature (Ehrlich and Miller, 1973). However, the decay rates of *B. subtilis* cells were not significantly influenced by the environmental temperature. In this study, we showed that high temperature (35°C) and lower RH (35%) have a synergistic effect on the decay of *Pal* in aerosols, with a half-life of 1.98 min; meanwhile, lower temperatures (25°C) and high RH (95%) support prolonged survival of *Pal* in aerosols, corresponding to a half-life of 6.79 min. This result suggested that high RH and low temperature favor the survival of *Pal* in aerosols.

There is strong evidence that temperature and RH affect the size of airborne particles, e.g., the size distributions of *B. subtilis* aerosols but not *P. fluorescens* aerosols were affected by RH (Johnson, 1999; Jones and Harrison, 2004; Pyankov et al., 2018). In this study, *Pal* aerosols with sizes of 0.65-2.1 μm were also profoundly affected by RH, and the concentration of *Pal* in aerosols in the 0.65-2.1 μm size range was relatively higher even under dry conditions. Our result was consistent with previous research showing that bacteria in smaller-sized aerosols (0.56-1 μm) have a higher viability (50%-70%) (Zhang et al., 2019). In addition, the distribution of *Pal* aerosols at 35°C and 65% RH was mainly 2.1-3.3 μm, which was similar to the *P. glyinea* aerosols collected in infected soybean plots during rainstorms and sprinkler irrigation (Venette and Kennedy, 1975). The stomatal aperture of healthy cucumber leaves is 4-5 μm (Wang et al., 2012), and the particle size of *Pal* aerosols released by cucumber plants predominantly ranged from 1.1 to 4.7 μm (accounting for 72.06%), which might be suitable for *Pal* aerosols to enter the cucumber plant and cause ALS disease (Chai et al., 2020). In this study, the particle size of *Pal* aerosols ranging from 2.1 to 4.7 μm was highest at 25°C and 95% RH. Interestingly, it is also the optimal condition for cucumber ALS disease development. Therefore, *Pal* aerosols distributed in the 2.1-4.7 μm range may play an important role in infecting cucumber plants.

High RH has a strong influence on the development of phyllosphere bacterial disease (Xin et al., 2016), and temperature impacts plant defense and bacterial virulence (Huot et al., 2017). In this study, the infectivity of *Pal* in aerosols to cucumber was significantly influenced by temperature and RH, and the highest infectivity of *Pal* in aerosols to cucumber was also shown at 25°C and 95% RH, in which the half-life of *Pal* in aerosols was much longer; this is similar to the findings of previous studies (Umekawa and Watanabe, 1980; Umekawa and Watanabe, 1982). Therefore, we consider 25°C and 95% RH as the optimal conditions for *Pal* to be infective to cucumber. The infectivity of *Pal* in aerosols is the lowest at 35% RH, which might be an extreme condition for *Pal* to infect cucumber. It is important to note that, however, the difference in the infectivity of *Pal* in aerosols to cucumber under various conditions was also affected by the concentration, survival and *Pal* in aerosols and the size of these aerosols, which needs more study to confirm.

## 5 Conclusions

This study investigated the effect of temperature and RH on the survival, release and infectivity of airborne *Pal* in aerosols. The release amount and survival time of *Pal* in aerosols under high RH (95%) and low temperature (≤ 25°C) conditions were significantly higher than those under low RH (35%) and high temperature (35°C) conditions. The highest release amount (96 CFU/m^3^) of *Pal* aerosol from infested cucumber and the survival rate (98.41%) were found at 18°C and 95% RH, while the highest disease index caused by *Pal* aerosol was found at 25°C and 95% RH. Additionally, the lowest amount of *Pal* released in aerosols and the fastest decay rate were detected at high temperature (35°C) and low RH (35%), and *Pal* aerosols in this condition resulted in the lowest disease index. These data provide valuable insight into the outbreaks of cucumber ALS disease and will aid further research into plant bacterial disease epidemiology by aerosol transmission. Our findings further suggest a novel means of preventing and controlling cucumber ALS disease by controlling the temperature and RH in greenhouses.

## Data availability statement

The original contributions presented in the study are included in the article/**Supplementary Material**. Further inquiries can be directed to the corresponding author.

## Author contributions

AC: Supervision, Funding acquisition, Methodology, Writing - review & editing. LY: Writing - original draft, Methodology, Resources. XL: Validation. LL: Formal analysis. YS: Data curation. XX: Investigation. BL: Conceptualization, Project administration. All authors contributed to the article and approved the submitted version.
