# Supplementary material for: Effect of temperature and humidity on dynamics and transmission of Pseudomonas amygdali pv. lachrymans aerosols
Source: Front Plant Sci. 2023 Feb 3;14:1087496. doi: 10.3389/fpls.2023.1087496 (PMC9936812; doi:10.3389/fpls.2023.1087496)
Supplement: Supplementary file 1 [file Table_1.docx]

**Table S1** Concentration of *Pseudomonas amygdali* pv. *lachrymans* (*Pal*) in aerosols in naturally infested cucumber greenhouses.

| Location | Greenhouse No. | Sampling  time | Temperature and  relative humidity | Concentration  of *Pal* aerosol  (cells/m^3^) |
| --- | --- | --- | --- | --- |
| Wuqing district,  Tianjin city | Ⅰ  (116.96 °E, 39.43 °N) | 2021.02.05 | 22.5°C, 92.0% RH | 876 ± 59 |
|  |  | 2021.02.06 | 19.7°C, 92.3% RH | 892 ± 95 |
|  |  | 2021.02.07 | 19.0°C, 91.5% RH | 826 ± 69 |
|  | Ⅱ  (116.84 °E, 39.41 °N) | 2019.02.15 | 31.4°C, 30.2% RH | 33 ± 7 |
|  |  | 2019.02.16 | 29.3°C, 40.9% RH | 40 ± 2 |
|  |  | 2019.02.17 | 30.7°C, 32.8% RH | 27 ± 3 |
| Guantao county,  Hebei province | Ⅲ  (115.24 °E, 36.60 °N) | 2020.12.29 | 23.6°C, 76.6% RH | 109 ± 18 |
|  |  | 2020.12.30 | 18.3°C, 73.9% RH | 91 ± 6 |
|  |  | 2020.12.31 | 20.1°C, 68.7% RH | 106 ± 21 |
|  | Ⅳ  (115.23 °E, 36.60 °N) | 2021.01.03 | 18.8°C, 69.8% RH | 173 ± 16 |
|  |  | 2021.01.04 | 17.0°C, 70.3% RH | 198 ± 29 |
|  |  | 2021.01.05 | 16.7°C, 75.9% RH | 215 ± 19 |
|  | Ⅴ  (115.23 °E, 36.61 °N) | 2019.02.10 | 19.0°C, 93.2% RH | 825 ± 68 |
|  |  | 2019.02.11 | 20.5°C, 95.1% RH | 900 ± 56 |
|  |  | 2019.02.12 | 21.2°C, 92.3% RH | 906 ± 50 |
| Shouguang county, Shandong province | Ⅵ  (117.62 °E, 35.78 °N) | 2020.11.06 | 21.7°C, 93.6% RH | 850 ± 95 |
|  |  | 2020.11.07 | 23.1°C, 91.5% RH | 955 ± 75 |
|  |  | 2020.11.08 | 22.4°C, 91.8% RH | 939 ± 93 |
|  | Ⅶ  (118.94 °E, 36.93 °N) | 2019.11.12 | 17.0°C, 92.1% RH | 793 ± 71 |
|  |  | 2019.11.13 | 18.6°C, 93.6% RH | 761 ± 62 |
|  |  | 2019.11.14 | 16.7°C, 94.9% RH | 740 ± 76 |
|  | Ⅷ  (119.22 °E, 36.74 °N) | 2021.01.07 | 18.1°C, 63.9% RH | 98 ± 19 |
|  |  | 2021.01.08 | 18.3°C, 62.6% RH | 80 ± 17 |
|  |  | 2021.01.09 | 18.8°C, 62.8% RH | 65 ± 12 |
|  | Ⅸ  (119.26 °E, 36.75 °N) | 2020.11.13 | 29.3°C, 40.5% RH | 61 ± 14 |
|  |  | 2020.11.14 | 29.6°C, 39.8% RH | 59 ± 9 |
|  |  | 2020.11.15 | 29.9°C, 35.1% RH | 45 ± 7 |

The concentration of *Pal* in aerosols was detected by qPCR, which are expressed as means ± standard deviations (SDs) of three replicates. Temperature and relative humidity in greenhouses were obtained using a digital thermo-hygrometer.
