# Supplementary material for: Effect of temperature and humidity on dynamics and transmission of Pseudomonas amygdali pv. lachrymans aerosols
Source: Front Plant Sci. 2023 Feb 3;14:1087496. doi: 10.3389/fpls.2023.1087496 (PMC9936812; doi:10.3389/fpls.2023.1087496)
Supplement: Supplementary file 2 [file DataSheet_1.docx]

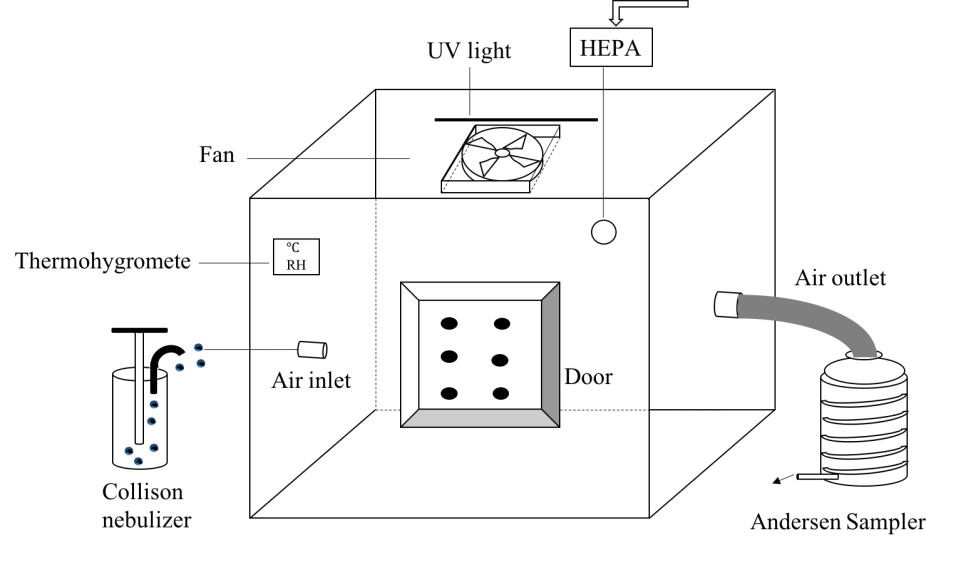


|  |
| --- |
|  |
| **Fig. S1** Schematic drawing of the aerosol chamber (length × width × height, 70 cm × 60 cm × 60 cm). The collision nebulizer and the Andersen sampler were connected to the air inlet and outlet, which were located on the left and right sidewalls of the aerosol chamber, respectively. A fan and a UV light were installed at the top wall for mixing the *Pal* aerosol well inside the chamber and sterilization, respectively. The door was fixed at the front wall with six screws, and a silicone pad was applied to ensure that the chamber was airtight. |
