# Supplementary material for: Effect of temperature and humidity on dynamics and transmission of Pseudomonas amygdali pv. lachrymans aerosols
Source: Front Plant Sci. 2023 Feb 3;14:1087496. doi: 10.3389/fpls.2023.1087496 (PMC9936812; doi:10.3389/fpls.2023.1087496)
Supplement: Supplementary file 3 [file DataSheet_2.docx]

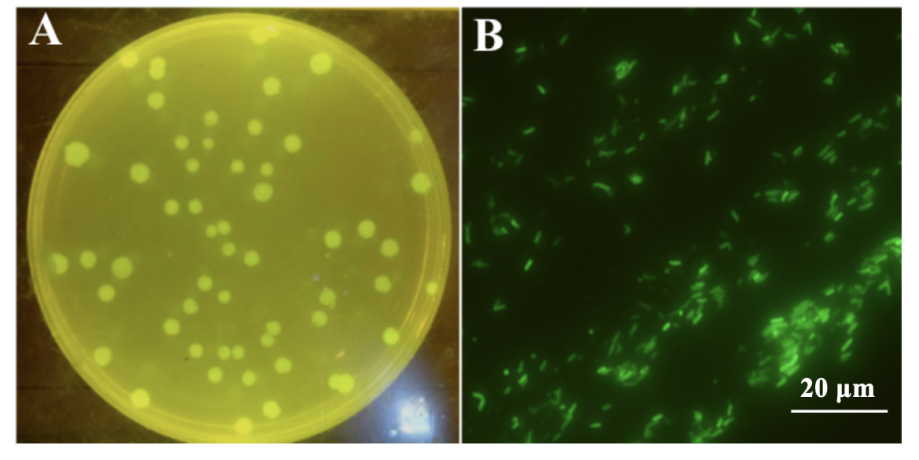


**Fig. S2** Fluorescence microscopy analysis. (A) *Pal*::GFP aerosol colonies on nutrient agar (NA) plates. (B) *Pal*:: GFP cell on cucumber diseased leaves.
